# A Role for Soluble ST2 in Vascular Remodeling Associated with Obesity in Rats

**DOI:** 10.1371/journal.pone.0079176

**Published:** 2013-11-12

**Authors:** Ernesto Martínez-Martínez, María Miana, Raquel Jurado-López, Elodie Rousseau, Patrick Rossignol, Faiez Zannad, Victoria Cachofeiro, Natalia López-Andrés

**Affiliations:** 1 Department of Physiology, School of Medicine, Universidad Complutense, Instituto de Investigación Sanitaria Gregorio Marañón (IiSGM), Madrid, Spain; 2 INSERM, Centre d’Investigations Cliniques- 9501, UMR 1116, Université de Lorraine and CHU de Nancy, Vandoeuvre-lès-Nancy, France; 3 Cardiovascular Translational Research. NavarraBiomed (Fundación Miguel Servet). Pamplona, Spain; I2MC INSERM UMR U1048, France

## Abstract

**Background:**

The function of the Interleukin-33 (IL-33)/ST2 system has been mainly investigated on immunological aspects, but recent data suggest that this pathway plays also an important role in cardiovascular system and adipose tissue. Whereas IL-33 has been demonstrated to exert anti-inflammatory and protective effects, circulating soluble ST2 (sST2) has emerged as a prognostic biomarker in patients with myocardial infarction and heart failure. Furthermore, sST2 is increased in severe obesity, although its role in the pathogenesis of vascular remodeling associated with obesity is still not well defined.

**Methodology/Principal Findings:**

Male Wistar rats fed standard diet (Control) or high fat diet (HFD) for 6 weeks. Aortic tunica media from diet-induced obese animals showed hypertrophy and fibrosis. The IL-33/ST2 system was spontaneously expressed in the aorta from Wistar rats. Administration of HFD in animals did not modify IL-33 expression at the transcriptional level. By contrast, HFD group showed an increase in aortic soluble sST2 and a decrease in the transmembrane isoform (ST2L) levels, resulting in decreased protective pathway activity. Aortic sST2 mRNA levels were associated with parameters showing vascular hypertrophy and fibrosis. *In vitro* experiments showed that primary cultured vascular smooth muscle cells (VSMCs) spontaneously expressed the IL-33/ST2 system. VSMCs stimulated with sST2 showed an increase in collagen type I, fibronectin and profibrotic factors.

**Conclusions:**

This is the first study demonstrating a deleterious role for sST2 in the vascular remodeling associated with obesity. In addition, we demonstrated that sST2 may act not only as a decoy receptor by binding IL-33 and preventing ST2L, but also modulating ECM remodeling and turnover. Thus, sST2 could be a new therapeutic target to reduce vascular remodeling in the context of obesity.

## Introduction

Health problems related to excess body weight have reached the dimensions of a pandemia in Western societies [1]. In particular, obesity has led to an increase in morbidity and mortality due to cardiovascular diseases [2]. Vascular structural alterations take place in the environment of obesity. Clinical and experimental studies have demonstrated that increases in body mass index are frequently associated with arterial stiffness and arterial wall thickness [3]. This involved direct effects on vascular smooth muscle cells (VSMCs) [4], the generation of reactive oxygen species, and the activation of nuclear factor κB (NFκB), which acts to stimulate growth and proliferation of VSMCs [5]. The increase in adipose tissue is associated with an aberrant secretion of adipokines and other vasoactive factors in adipose tissue which is a major contributor to the onset and progression of obesity-related vascular complications affecting extracellular matrix (ECM) turnover. However, the mechanisms by which obesity induces vascular remodeling have not been fully elucidated.

ST2, also designated as T1, Fit-1 or DER-4, is an interleukin-1 receptor family member that was originally described as a gene induced by serum stimulation of fibroblasts [6], [7]. ST2 gene encodes at least 3 isoforms of ST2 proteins by alternative splicing: ST2L, a transmembrane isoform; a secreted soluble ST2 (sST2) form that lacks the transmembrane and intracellular domains, and ST2V, a variant form present mainly in the gut of humans [8]. The transmembrane ST2 isoform (ST2L) is a membrane-bound isoform with 3 extracellular IgG domains, a single transmembrane domain, and an intracellular SIR domain homologous to TLRs and other IL-1Rs [9]. Soluble ST2 is identical to the extracellular region of the long ST2 isoform except for nine additional amino acids, which are present at the C terminus of the molecule [10]. Once Interleukin-33 (IL-33) binds ST2L [11] sequesters the adaptor protein myeloid differentiation factor 88 (MyD88), resulting in interleukin-1 receptor-associated kinase 1 (IRAK-1), mitogen-activated protein kinase (MAPK) and NFκB modulation. IL-33 appears to be a cytokine with dual function, acting both as a traditional cytokine and as an intracellular nuclear factor with transcriptional regulatory properties [12]. The most protective actions of IL-33 are attributed to ST2L and sST2 has been considered simply as a decoy receptor to prevent IL-33 binding to and signaling through ST2L [9].

The expression of the components of the IL-33/ST2 system has been reported in many tissues, including myocardium [13], coronary artery endothelium [14], coronary vessels [15] and adipose tissue [16]. The function of the IL-33/ST2 system has been mainly investigated on immunological aspects, but recent data also suggest that the IL-33/ST2 pathway plays an important role in the cardiovascular system. Circulating sST2 has emerged as a prognostic biomarker in patients with myocardial infarction and heart failure [17]–[19]. Furthermore, sST2 is increased in severe obesity [16], although its role in the pathogenesis of vascular remodeling associated with obesity is still unknown.

The relevance of this ligand-receptor system to physiological or pathological function of vascular smooth muscle cells (VSMCs) is unknown at this time. The aim of this study was to highlight the expression and the effects of IL-33/ST2 system, and particularly of sST2 in VSMCs and to determine whether sST2 could be a new biotarget reducing vascular remodeling associated with obesity.

## Methods

### Animals

The investigation was performed in accordance with the Guide for Care and Use of Laboratory Animals published by the U.S. National Institutes of Health (publication no. 82-23, revised in 1996) and were approved by the local ethical committee “Comité regional Nancy-Lorraine/Nord-Est” (n° B54-547-20). Studies were performed in 150g male Wistar rats (Charles River, France). Rats were housed at constant room temperature (20–22°C), humidity (50–60%), with light cycle (12; 12-light-dark) with free access to a standard diet (3.5% fat; Harlan Teklad #TD.2014, n = 7) or a high fat diet (HFD, 33.5% fat; Harlan Teklad #TD.03307, n = 7) for 6 weeks. Body weight was measured once a week. Blood pressure (SBP) was estimated basally and at the end of the study by use of a tail-cuff plethysmograph (Narco Bio-Sustems) in unrestrained animals as previously reported [20]. For euthanasia, rats were anesthetized i.p. with a cocktail of ketamine (Imalgene 1000) 70 mg/kg and xilacine (Rompun 2%) 6 mg/kg.

### Vascular Smooth Muscle Cell Isolation and Culture

Rat aortic vascular smooth muscle cells (VSMC) were isolated from the thoracic aorta of male Wistar rats (250 gr body weight) as previously described [21]. The cells were maintained in DMEM medium supplemented with 10% FBS. All assays in the present study were done at temperatures of 37°C, 95% sterile air and 5% CO2 in a saturation humidified incubator. VSMCs were used between passages 6 and 7. For experiments, cells were seeded into 6-well plates at 90% confluence and serum starved for 12 h. Cells were then cultured in the same medium and stimulated with sST2 (2 µg/ml, R&D System) for 6 hours for mRNA determinations and for 24 hours for protein analysis.

### Aortic Composition

Aortic segments were opened longitudinally, the media separated from the adventitia and the medial length measured under a microscope. Media were then defatted, dried and weighed. Medial cell proteins were extracted by 0.3% sodium dodecyl sulfate (SDS) and subsequently assayed, insoluble elastin was purified by the hot alkali method and quantified by weighing. Proteins in the NaOH extract were then hydrolysed, and total medial collagen was quantified by assaying hydroxyproline in the hydrolysate, using a colorimetric assay, as previously reported [21].

### Morphological and Histological Evaluation

Aorta segments were rapidly cleaned from the surrounding tissues and blood, cut into rings fixed in formalin 37% and embedded in paraffin blocks. Afterwards, five micrometer thick sections were cut with a rotational microtome (Leitz 1512, IMEB INC), placed onto glass microscope slides and stained with picrosirius red or hematoxylin and eosin by routine methods. Images from transverse sections of the arterial segments were captured using a camera connected to an optical microscope. Media and lumen areas were measured in triplicate and were quantified by planimetry using an image analyzer (LEICA Q550 IWB). In order to avoid miscalculation of the data due to possible deformation of the vessels during their preparation, we have determined luminal or vessel area by correcting the cross-sectional area enclosed by the internal or external elastic lamina, respectively, to a circle by applying the form factor l2/4Π to the measurement of the lamina, where l is the length of the lamina. Media/Lumen ratio was calculated from area data. Media thickness of the aorta was measured in eight-ten different regions with LEICA software.

### Immunohistological and Immunocytochemistry Evaluation

Either paraffin-embedded aorta sections (5 µm) or VSMCs fixed in 4% PFA were used. Slides were treated with H_2_O_2_ for 10 min to block peroxidase activity. All samples were blocked with 5% normal goat serum in PBS for 1 h and incubated overnight with IL-33 (Santa Cruz Biotechnology, dilution 1∶50), ST2 (Novus Biologicals, dilution 1∶50), MyD88 antibodies (Santa Cruz Biotechnology, dilution 1∶50), washed three times, and then incubated for 30 min with the horseradish peroxidase-labeled polymer conjugated to secondary antibodies (Dako Cytomation). The signal was revealed by using DAB Substrate Kit (BD Pharmingen). As negative controls, samples followed the same procedure described above but in the absence of primary antibodies.

### Real Time Reverse-transcription PCR

Total RNA was extracted with Trizol Reagent (Euromedex) and purified using the RNeasy kit, according to the manufacturer’s instructions (Qiagen). First strand cDNA was synthesized according to the manufacturer’s instructions (Roche). Quantitative PCR analysis was then performed with SYBR green PCR technology (ABGene) (Table 1).

**Table 1:** Primers used in real time PCR analysis.

Gene	Primers	Sequence (5′ to 3′)
**Col I**	Forward	GCC TCC CAG AAC ATC ACC TA
	Reverse	ATG TCT GTC TTG CCC CAA GT
**Fibronectin**	Forward	GGG GTC ACG TAC CTC TTC AA
	Reverse	TGG AGG TTA GTG GGA GCA TC
**TGF-β**	Forward	CAG AAG TTG GCA TGG TAG CC
	Reverse	TGC TTC AGC TCC ACA GAG AA
**CTGF**	Forward	GAG TCG TCT CTG CAT GGT CA
	Reverse	CCA CAG AAC TTA GCC CGG TA
**IL-33**	Forward	TCG CAC CTG TGA CTG AAA TC
	Reverse	ACA CAG CAT GCC ACA AAC AT
**sST2**	Forward	CGTTACCTTCCTGTGCCATT
	Reverse	CTCCATTTGCCAATCATGTG
**ST2L**	Forward	AGT TGT GCA TTT ACG GGA GAG
	Reverse	GGA TAC TGC TTT CCA CCA CAG
**IL-6**	Forward	GCC CTT CAG GAA CAG CTA TG
	Reverse	GTC TCC TCT CCG GAC TTG TG
**OPN**	Forward	ATG AGA CTG GCA GTG GTT
	Reverse	GCT TTC ATT GGA GTT GCT
**MCP-1**	Forward	TTC CTT ATT GGG GTC AGC AC
	Reverse	CAG TTA ATG CCC CAC TCA CC
**HPRT**	Forward	AGG ACC TCT CGA AGT GT
	Reverse	ATT CAA ATC CCT GAA GTA CTC AT

Relative quantification was achieved with MyiQ (Bio-rad) software according to the manufacturer’s instructions. Data were normalized by HPRT levels and expressed as percentage relative to controls. All PCRs were performed at least in triplicate for each experimental condition.

### Western Blot Analysis

Aortas were dissected, cut longitudinally (to remove the endothelium), and the adventitia layer was removed. Then aortic tunica medias were immediately frozen in liquid nitrogen for molecular studies. Aliquots of 20 µg of proteins from aortas and VSMCs were electrophoresed on SDS polyacrylamide gels and transferred to Hybond-c Extra nitrocellulose membranes (Amersham Biosciences). Membranes were incubated with primary antibodies for : collagen type I (Biogenesis, dilution 1∶500), fibronectin (Millipore, dilution 1∶500), elastin (Abcam, dilution 1∶250), connective tissue growth factor (CTGF; Sigma, dilution 1∶1000), transforming growth factor β (TGF-β; Abcam, dilution 1∶500), IL-33 (Santa Cruz Biotechnology, dilution 1∶500), ST2 (Novus Biologicals, dilution 1∶500), myeloid differentiation primary-response protein–88 (MyD88; Santa Cruz Biotechnology, dilution 1∶250), IRAK-1 (Santa Cruz Biotechnology, dilution 1∶250), and β-actin (Sigma, dilution 1∶1000) as a loading control. After washing, detection was made through incubation with peroxidase-conjugated secondary antibody, and developed using an ECL chemiluminescence kit (Amersham). After densitometric analyses, optical density values were expressed as arbitrary units. Results are expressed as an n-fold increase over the values of the control group in densitometric arbitrary units. All Western Blots were performed at least in triplicate for each experimental condition.

### Gelatin Zymography

Aliquots of culture media containing 30 µl of supernatant were resolved on a 10% SDS polyacrylamide gel containing 0.3% gelatin. The gel was rinsed 3 times for 15 min with a solution of 2.5% Triton X 100 to remove SDS and renature the proteins, followed by incubation for 48 h at 37°C in 1 mol/L Tris-HCl, pH 7.5 with 1 mol/L CaCl_2_, and 5 mol/L NaCl to promote degradation of gelatin. Gels were fixed in 40% methanol and 10% acetic acid, and then stained for 30 min in 0.25% Coomassie blue R-250 to identify proteolytic activity of MMPs.

### Statistical Analysis

Data are expressed as mean±SEM. Normality of distributions was verified by means of the Kolmogorov-Smirnov test. Pearson correlation analysis was used to examine association among different variables. Body weight and blood pressure were analyzed using a two-way analysis of variance, followed by a Bonferroni test. The rest of the data were analyzed using an unpaired t test, using GraphPad Software Inc. (San Diego, CA, USA). The predetermined significance level was α = 0.05.

## Results

### Effect of High Fat Diet on Vascular Fibrosis and Inflammation in Rats

**Figure 1:**
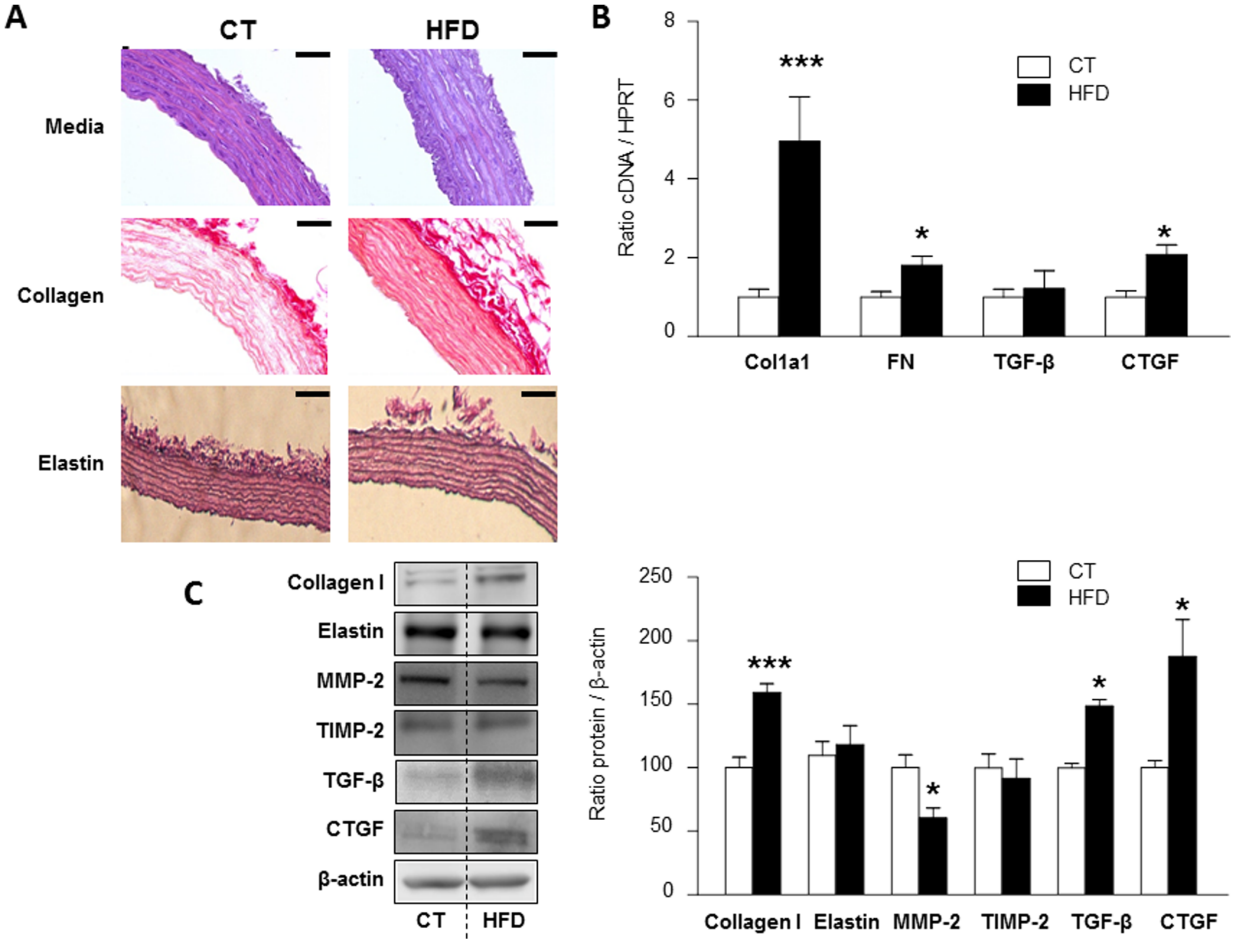
Aortic composition in HFD rats. Aortas from rats fed a standard diet (3.5% fat) or a high fat diet (HFD, 33.5% fat) were analyzed. Representative pictures of slides stained for collagen and elastin are presented (magnification 40X) (**A**). mRNA expression of collagen type I, fibronectin, TGF-β, CTGF in aorta from controls and HFD rats (**B**). Protein expression of collagen type I, elastin, MMP-2, TIMP-2, TGF-β and CTGF in aorta from controls and HFD rats (**C**). All conditions were performed at least by triplicate. Scale bar 50 µm. Histogram bars represent the mean ± SEM of 6–7 animals, in arbitrary units normalized to HPRT and β-actin for cDNA and protein respectively. *p<0.05; ***p<0.001 *vs*. control group.

From the first week onwards, body weight gain was significantly higher in rats fed a high fat diet (HFD) as compared with rats fed a standard diet (data not shown), the relative increase in body weight at the end of the experiment being 33% in HFD as compared to the control group (Table 2). Importantly, blood pressure levels did not change in both groups (Table 2). Diet-induced obese animals showed important changes in the aortic wall (Table 3). Aortic dry weight and cell protein content were significantly increased by the high fat diet as compared with control rats. There was a significant increase in collagen to elastin ratio in HFD group. This increase was due to an augmented collagen levels without changes in aortic elastin content. These changes were accompanied by morphometric modifications. There was a significant increase in media to lumen ratio in HFD group, due to an augmented media area without changes in lumen area. These results were confirmed by histological techniques, as shown in Figure 1A.

**Table 2:** Evolution of body weight and systolic blood pressure in controls and HFD rats.

	Control		HFD	
**Week**	0	6	0	6
**BW (g)**	165.16±3.361	317.73±10.018#	168.31±3.364	371.46±8.299** #
**SBP** **(mmHg)**	120.18±3.523	116.35±5.785	124.78±3.376	121.010±2.481

BW: Body weight; SBP: Systolic blood pressure. Values are mean ± SEM,

** p<0.01 *vs*. control group;

# p<0.01 *vs.* week 0.

**Table 3:** Parameters measured in aortic tunica media from Controls and HFD rats.

	Control	HFD
**n**	6	6
**Dry weight**	2.01±0.099	2.21±0.059*
**Cell proteins**	0.180±0.033	0.400±0.172*
**Elastin**	56.46±1.646	57.82±1.233
**Collagen**	5.11±0.715	10.06±0.671***
**Collagen/Elastin**	0.09±0.033	0.17±0.029***
**Lumen area (mm^2^)**	1.53±0.07	1.44±0.05
**Media Area (mm^2^)**	0.42±0.01	0.51±0.01**
**Media/Lumen ratio**	0.28±0.01	0.33±0.008**
**Media thickness (µm)**	96.6±1.07	115.4±5.1**
**IL-6**	1.00±0.35	3.98±0.70*
**OPN**	1.00±0.46	17.52±4.30**
**MCP-1**	1.00±0.21	4.60±0.46***

Dry weight and contents in proteins are expressed in mg/cm. Parameters of inflammation were performed at least by triplicate and expressed in arbitrary units normalized to HPRT for cDNA. Values are mean ± SEM.

* p<0.05;

** p<0.01;

*** p<0.001 *vs*. control group.

Complementary assays to analyze in depth the molecular alterations that accompanied hypertrophy, fibrosis and inflammation in aorta from HFD animals were performed. mRNA analysis revealed that HFD increased aortic interleukin-6 (IL-6; 3.9-fold; p<0.05), osteopontin (OPN; 17.5-fold; p<0.01) and monocyte chemoattractant protein-1 (MCP-1; 4.6-fold; p<0.001) (Table 3). These changes were accompanied with modifications on vascular fibrosis in collagen type I (4.9-fold; p<0.001), and fibronectin (1.8-fold; p<0.05) synthesis (Figure 1B). Moreover, the profibrotic factor CTGF was increased (2.1-fold; p<0.05) in aorta from HFD group, without changes in TGF-β mRNA levels (Figure 1B).

At the protein level, HFD animals showed an increase in aortic protein expression of collagen type I (1.6-fold; p<0.001). This increase was not observed in elastin protein levels (Figure 1C). In addition, HFD rats showed a reduction of MMP-2 protein levels (0.6-fold; p<0.05) but not changes in one of its inhibitors, TIMP-2, suggesting a reduction in its activity and in consequence, less collagen degradation as compared with control animals (Figure 1C). Likewise, HFD animals presented higher levels of profibrotic factors TGF-β (1.5-fold; p<0.05) and CTGF (2.1-fold; p<0.05) (Figure 1C).

### Effect of High Fat Diet on Aortic IL-33/ST2 Pathway

**Figure 2:**
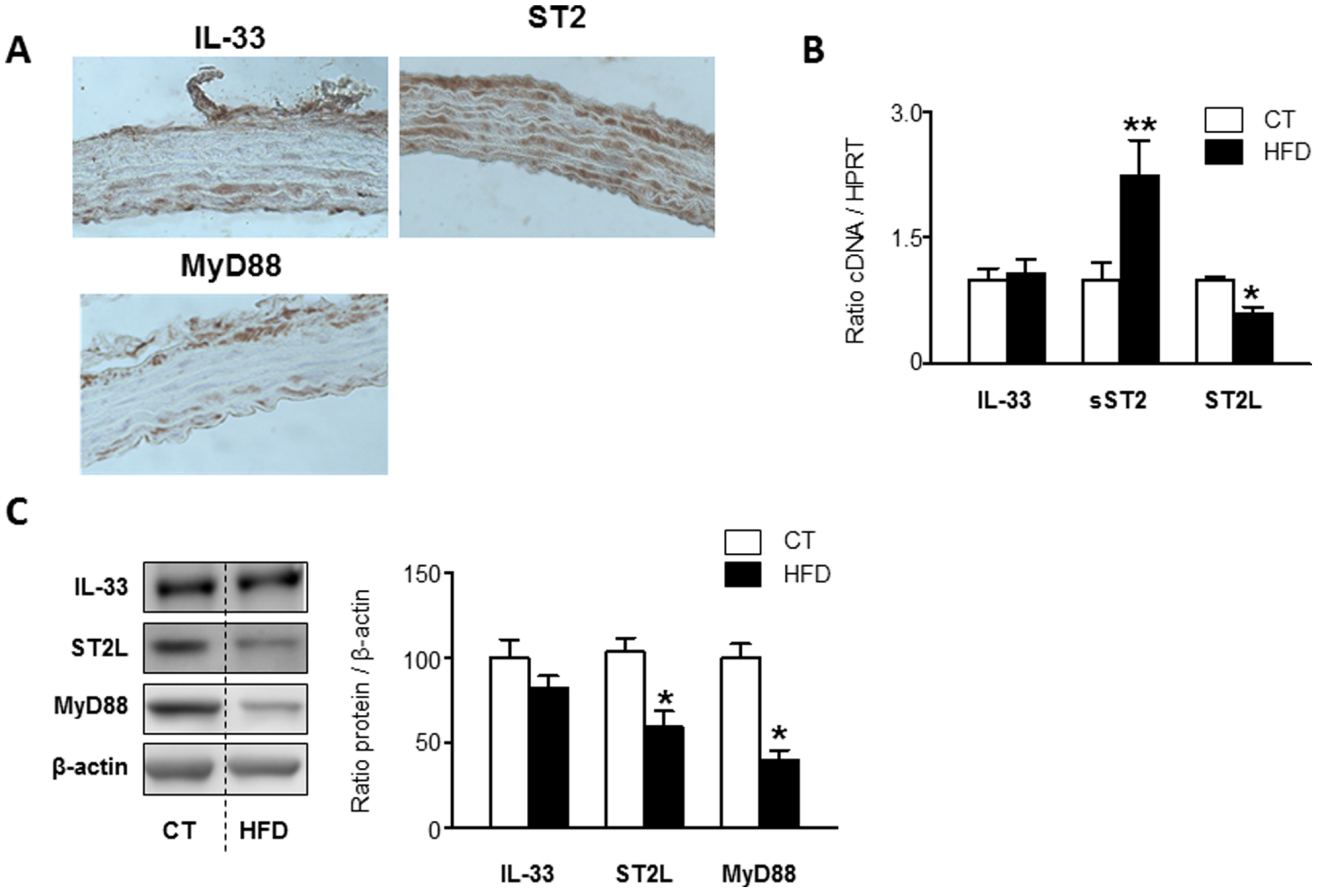
Effect of high fat diet on the IL-33/ST2 pathway in the aorta of rats. Representative pictures of slides immunostained for IL-33, ST2 and MyD88 are presented (**A**). Aortic mRNA levels of IL-33, sST2 and ST2L (**B**). Protein levels of aortic IL-33, ST2L and MyD88 are shown (**C**). All conditions were performed at least by triplicate. Histogram bars represent the mean ± SEM of 6–7 animals, in arbitrary units normalized to HPRT and β-actin for cDNA and protein respectively. *p<0.05; **p<0.01 *vs*. control group.

As shown in Figure 2A, the IL-33/ST2 system was spontaneously expressed in the aorta from Wistar rats. ST2 was expressed in the adventitia, the media and the intima layers. IL-33 and MyD88 were mainly expressed in the adventitia and the intima layers.

Administration of HFD in animals did not modify IL-33 expression at the transcriptional level. By contrast, HFD group showed an increase in sST2 (2.2-fold; p<0.01) and a decrease in ST2L levels (0.6-fold; p<0.05) (Figure 2B).

Similarly, HFD animals did not show any modifications in aortic protein levels of IL-33, but showed a decrease in protein levels of ST2L (0.6-fold; p<0.05) and MyD88 (0.4-fold; p<0.05) (Figure 2C).

Direct correlations in all animals were found among arterial sST2 mRNA levels and parameters showing hypertrophy and fibrosis in the aorta. Aortic sST2 mRNA levels were strongly correlated with cell protein content (r^2^ = 0.7469; p = 0.0006), vascular collagen type I mRNA expression (r^2^ = 0.7818; p<0.0036), vascular collagen type I protein expression (r^2^ = 0.5226; p<0.012), vascular CTGF mRNA expression (r^2^ = 0.6359; p<0.01) and vascular CTGF protein expression (r^2^ = 0.4506; p<0.0477).

### Effects of sST2 on IL-33/ST2 System in Isolated Vascular Smooth Muscle Cells

**Figure 3:**
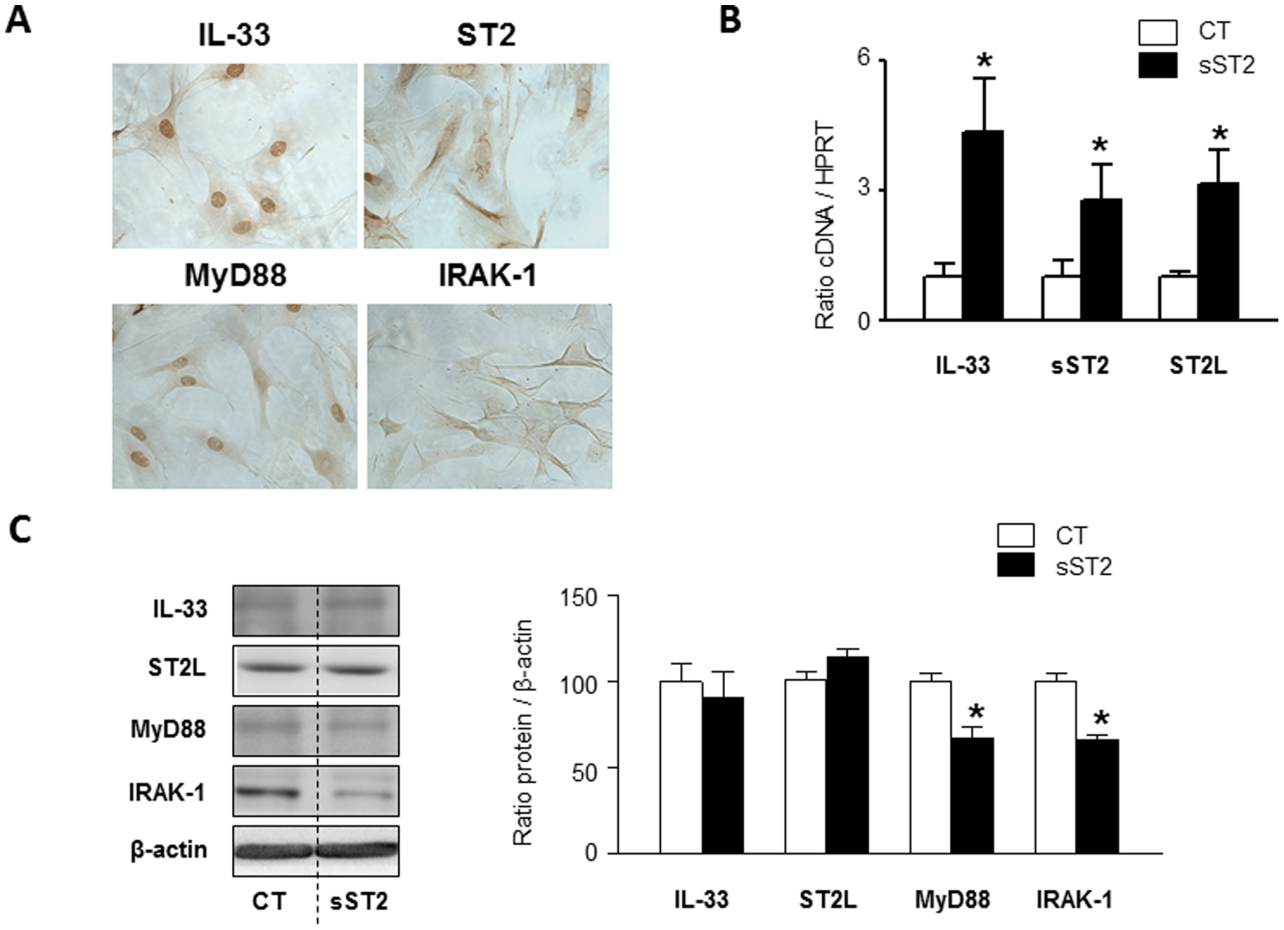
Effect of sST2 on the IL-33/ST2 pathway in VSMCs. Representative pictures of immunocytochemistry for IL-33, ST2, MyD88 and IRAK-1 are presented (**A**). mRNA levels of IL-33, sST2 and ST2L in VSMCs stimulated with sST2 (2 µg/ml) (**B**). Protein levels of IL-33, ST2-L, MyD88 and IRAK-1 are shown (**C**). All conditions were performed at least by triplicate. Histogram bars represent the mean ± SEM of 4 assays, in arbitrary units normalized to HPRT and β-actin respectively for cDNA and protein. *p<0.05 *vs*. control.

As shown in Figure 3A, VSMCs spontaneously expressed IL-33/ST2 system. Nuclear localization for IL-33 and MyD88 was found, whereas ST2 and IRAK-1 were located in the cytoplasm of VSMCs, mainly in the perinuclear region.

We next investigated whether sST2 could be involved in the regulation of the IL-33/ST2 system in VSMCs. Cells stimulated with sST2 presented increased IL-33 (4.3-fold; p<0.05), sST2 (2.8-fold; p<0.05) and ST2L (3.1-fold; p<0.05) mRNA expressions (Figure 3B). In contrast, in the protein analysis, sST2 stimulated cells did not show modifications in IL-33 neither ST2 levels, but showed a decrease in MyD88 (0.6-fold; p<0.05) and IRAK-1 (0.6-fold; p<0.05) levels (Figure 3C).

### Profibrotic Effects of sST2 in Vascular Smooth Muscle Cells

In order to investigate whether the increase in aortic sST2 observed in HFD animals could have a pathophysiological relevance in vascular fibrosis, we analysed the effects of sST2 in VSMCs and the molecular mechanism involved.

**Figure 4:**
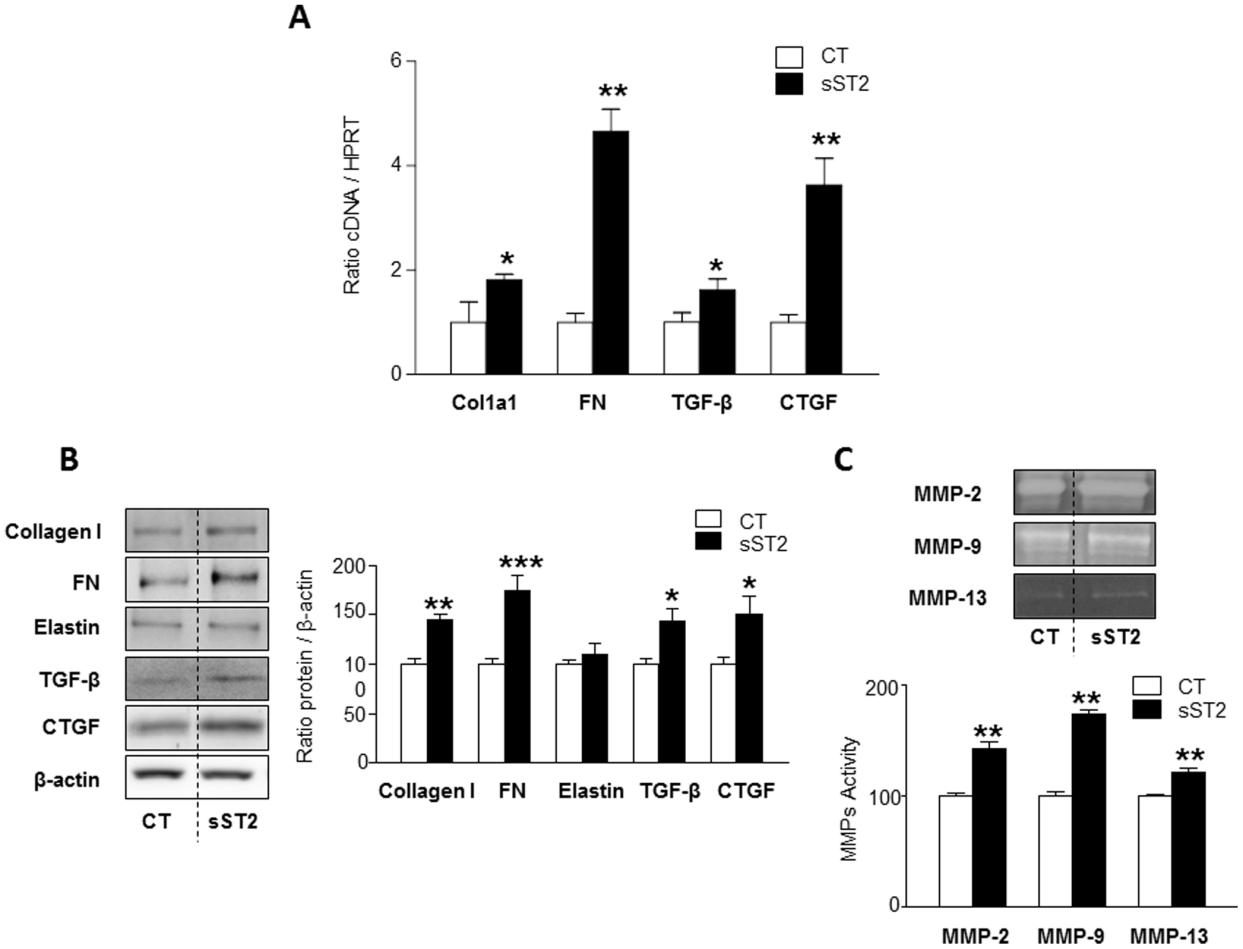
Effects of sST2 on fibrotic parameters in VSMCs. mRNA levels of collagen type I, fibronectin, TGF-β and CTGF in VSMCs stimulated with sST2 (2 µg/ml) during 6 hours (**A**). Protein expression of collagen type I, fibronectin and elastin (**B**). MMP-2, MMP-9, MMP-13 activity in VSMCs stimulated with sST2 (**C**). All conditions were performed at least by triplicate. Histogram bars represent the mean ± SEM of 4 assays, in arbitrary units normalized to HPRT and β-actin respectively for cDNA and protein. *p<0.05; **p<0.01; ***p<0.001 *vs*. control.

Incubation with sST2 increased mRNA expression of collagen type I (1.8-fold; p<0.05), fibronectin (4.6-fold; p<0.01), and different profibrotic factors such as TGF-β (1.6-fold; p<0.05) and CTGF (3.6-fold; p<0.05) (Figure 4A).

Furthermore, sST2 increased protein expressions of collagen type I (1.5-fold; p<0.01), fibronectin (1.7-fold; p<0.001), TGF-β (1.5-fold; p<0.05), CTGF (1.5-fold; p<0.05) but not elastin (Figure 4B).

Analysis of conditioned media by gelatin zymography revealed that sST2 enhanced the proteolytic activity of the bands corresponding to active MMP-2 (1.4-fold; p<0.01), MMP-9 (1.7-fold; p<0.01) and MMP-13 (1.3-fold; p<0.01) (Figure 4C).

## Discussion

The purpose of this study was to investigate the involvement of the IL-33/ST2 system in arterial wall remodeling associated with obesity. We here demonstrated that the IL-33/ST2 pathway was spontaneously expressed in aorta and in primary cultured VSMCs. Furthermore, the expression of sST2 was up-regulated in aorta from obese animals and associated to parameters showing vascular hypertrophy and fibrosis. Moreover, our study shows for the first time that sST2 enhances ECM production in vascular cells. These results suggest that the IL-33/ST2 could be a new key pathway involved in arterial wall remodeling associated with obesity.

Normotensive diet-induced obese animals showed aortic wall remodeling characterized by an increase in media cross-sectional area and wall thickness. These morphological alterations were accompanied by increased in inflammation parameters (IL-6, OPN and MCP-1) and ECM proteins deposition (mainly collagen type I and fibronectin) in the absence of elastin modifications, supporting increased arterial stiffness in the obese animals, a change known to be predictive of increased cardiovascular mortality [22], [23]. According to our experimental data, and also independently from the presence of hypertension and diabetes, obese patients present increased media-to-lumen ratio and increased media cross-sectional area of subcutaneous small resistance arteries [24]. Moreover, some data suggests that body weight and fat distribution are also related to arterial stiffening. In this way Sutton-Tyrrel and col. found that among healthy older individuals, measures of body weight and degree of fat were correlated with greater vascular stiffness [25]. Obesity associated vascular remodeling is characterized by a lasting change in the diameter of blood vessels that requires coordinate degradation and deposition of ECM to preserve the general architecture of the arterial wall. The dysregulation of the balance between aortic MMP-2 and its inhibitor TIMP-2 observed in HFD animals may contribute to degrade the ECM components by creating uncoiled, less effective collagen and broken and frayed elastin molecules [26]. In addition we observed an increase in profibrotic factors like TGF-β together with its downstream mediator, CTGF in HFD animals as compared with control rats that could mediate the increase in collagen production observed in obese animals. TGF-β has long been believed to be the most important ECM regulator and it plays an important role in maintaining vessel wall structure and controls the balance between inflammation and ECM deposition [27]. These data are in agreement with previous studies from our group showing that CTGF is directly involved in vascular remodeling associated with hypertension [28].

It is generally believed that the composition of the ECM is a critical determinant of arterial stiffness, but the signaling pathways involved in this process are relatively underexplored [29] and so it is necessary to establish new targets to manipulate it therapeutically. In this line, we investigated the role of the IL-33/ST2 pathway as a mediator of obesity-induced ECM remodeling. It has been reported that mRNA of IL-33 and ST2L are present in thoracic aorta from mice and in human VSMCs [30]. However, the authors did not detect the protein IL-33 in the media of large vessels. Another recent study reported that a weak expression of the protein IL-33 is observed in human arteries, more precisely in VSMCs, whereas ST2L is only expressed in endothelial cells [31]. However, to our knowledge, there are no studies analysing the expression of sST-2 neither in the vascular wall nor in VSMCs. In the present study, we clearly demonstrated by three different techniques (real time RT-PCR, Western Blot and immonohistochemistry) the presence of IL-33, ST-2L and sST-2 in the vascular tunica media and in isolated primary cultured VSMCs, both at the mRNA and at the protein levels. The discrepancies between our results and previous studies could be due to the different tools used to highlight the expression of these three molecules. Of special interest, the soluble form of ST-2, sST-2, is also spontaneously expressed by VSMCs, suggesting a physiological role for this molecule in vascular function. The presence of sST2 in vasculature from control normotensive rats suggests that the molecule could play a role in physiological conditions binding to IL-33. sST2 act as a decoy regulating its function such a traditional cytokine and as an intracellular nuclear factor with transcriptional regulatory properties [12].

It is currently well-accepted that obesity promotes a state of chronic low-grade inflammation, reflected by an increased production of cytokines and proinflammatory adipokines by adipose tissue [32]. sST2 levels are increased in heart failure patients in response to cardiac stress but also in response to inflammation; in these conditions, sST-2 is released into the circulation [33]. While the lung has been shown to have the highest expression of sST2 [34], potential cellular sources of sST2 in the cardiovascular system include endothelial cells [14] and cardiac myocytes [34]. Furthermore, the secretory capacity of these cells for sST2 was greatly enhanced by certain pro-inflammatory cytokines (e.g. TNFα and IL-1β) and by supernatants derived from LPS-stimulated mononuclear cells [34].

The upregulation of sST2 has been described in adipose tissue from severe obesity patients, predominantly in endothelial cells [16]. However, its possible role in vascular remodeling has never been investigated. HFD animals presented an important increase in vascular sST2 accompanied by a decrease in ST2L while no changes were observed in IL-33. In addition, we observed a decrease in MyD88, involved in interleukin 1 receptor signaling through ST2L. These results suggest that in obesity IL-33 could lack its vascular protective properties linked to ST2L attributed by another authors [12], [35]. The positive correlation between sST2 and hypertrophic and fibrotic indices such as aortic cell proteins content, collagen and CTGF vascular levels observed in this study suggests that sST2 could play an important role in obesity-induced vascular remodeling. We demonstrate for the first time, that sST2s stimulates the synthesis of ECM by increasing collagen and fibronectin levels as well as the profibrotic molecules TGF-β and CTGF. Likewise sST2 is able to increase MMPs activities. Altogether these results suggest that sST2 is a new player in vascular fibrosis. In addition, VSMCs stimulated with sST2 showed a decrease in MyD88 and IRAK-1, without changes in IL-33 protein levels, corroborating the results found in the aortas of animals fed a HFD in which sST2 acts as a decoy receptor for IL-33. In accordance to these data, a recent study has shown that sST2 administration exacerbates atherosclerosis development in a mouse model of atherosclerosis by suppressing IL-33 function [30].

This is the first study demonstrating a modulatory role for sST2 in the vascular remodeling associated with obesity. We show that sST2 is increased in aorta from obese rats, and in addition is inducing vascular fibrosis, suggesting that sST2 could participate in the remodeling observed in obesity. sST2 may act not only as a decoy receptor by binding IL-33 and preventing ST2L signaling, but also exerts its direct effects modulating ECM remodeling and turnover. Further understanding of the molecular mechanisms by which sST2 regulates VSMCs function may lead to novel targeted therapies from vascular diseases associated to obesity.
